# CellMarkerPipe: cell marker identification and evaluation pipeline in single cell transcriptomes

**DOI:** 10.1038/s41598-024-63492-z

**Published:** 2024-06-07

**Authors:** Yinglu Jia, Pengchong Ma, Qiuming Yao

**Affiliations:** 1 School of Computing, University of Nebraska Lincoln, 256 Avery Hall, Lincoln, NE 68588 USA; 2 Department of Chemistry, University of Nebraska Lincoln, Hamilton Hall, Lincoln, NE 68588 USA; 3 Nebraska Center for the Prevention of Obesity Diseases, 316C Leverton Hall, Lincoln, NE 68583 USA; 4 Nebraska Center for Virology, University of Nebraska, 4240 Fair St., Lincoln, NE 68583 USA

**Keywords:** Computational platforms and environments, Data processing, Genome informatics, Software, Standards

## Abstract

Assessing marker genes from all cell clusters can be time-consuming and lack systematic strategy. Streamlining this process through a unified computational platform that automates identification and benchmarking will greatly enhance efficiency and ensure a fair evaluation. We therefore developed a novel computational platform, cellMarkerPipe (https://github.com/yao-laboratory/cellMarkerPipe), for automated cell-type specific marker gene identification from scRNA-seq data, coupled with comprehensive evaluation schema. CellMarkerPipe adaptively wraps around a collection of commonly used and state-of-the-art tools, including Seurat, COSG, SC3, SCMarker, COMET, and scGeneFit. From rigorously testing across diverse samples, we ascertain SCMarker’s overall reliable performance in single marker gene selection, with COSG showing commendable speed and comparable efficacy. Furthermore, we demonstrate the pivotal role of our approach in real-world medical datasets. This general and opensource pipeline stands as a significant advancement in streamlining cell marker gene identification and evaluation, fitting broad applications in the field of cellular biology and medical research.

## Introduction

Single-cell RNA sequencing (scRNA-seq) has emerged as a powerful high-throughput technique, enabling the comprehensive profiling of diverse cell populations within tissue samples^1–6^. The scRNA-seq technology not only facilitates the exploration of various biological processes in disease and development^7–9^, but also allows for the identification of both known and novel single cell types, along with the characterization of their respective marker genes^10–12^. In typical scRNA-seq analysis, following cell type clustering is to obtain marker genes that are specific to the clusters^13^. These marker genes are then manually inspected using available information in the literature or cell marker databases such as CellMarker^14^ and PanglaoDB^15^. While effective, this manual process can be time-consuming and potentially prone to biases when different marker gene identification approaches need to be tested and applied.

A range of computational tools has emerged to enhance the convenience and automation of marker gene identification in scRNA-seq analysis. ScType streamlines cell type annotation through a reference marker gene database^16^, emphasizing the crucial role of marker gene identification under the cluster context. General-purpose feature selection, particularly dimension reduction based on globally highly variable genes, retains informative genes but may not offer cell type markers. In contrast, for de-novo marker gene identification, methods targeting differential expression (DE) genes have been proven effective in pinpointing genes specific to cell types^17^. Through extensive testing among DE statistical approaches, Wilcoxon rank sum test was highlighted to be worked well for DE gene identification particularly with sufficient sample size^17–19^. Seurat, a cornerstone package in scRNA-seq, performs non-parametric Wilcoxon rank sum test by default in FindAllMarkers function^20^ in a one-against-all manner. SC3^21^, another comprehensive scRNA-seq analysis package, identifies DE genes through a non-parametric Kruskal–Wallis test.

Additional to DE based statistical tests, there exists a category of specialized tools with more sophisticated approaches for cluster-wise marker gene identification. These tools aim to computationally emulate cell sorting by identifying cell type-specific genes or gene panels. COSG^22^ presents a significant advancement in the field by introducing cosine similarity-based marker gene identification, which proves to be a more precise, robust and scalable method for discerning true marker genes across various cell types. SCMarker^23^ is an ab initio method designed for marker selection by exploring bi-modally distributed expression levels that are co- or mutually-exclusively expressed with some other genes. Then SCMarker assigns the top ten highest expressed genes from all markers to the specific cell types. Uniquely, COMET^24^ has capability in predicting advantageous marker panels (gene combinations) from transcriptomic data, by a special hypergeometric test. Finally, scGeneFit^25^ selects gene markers that collectively optimize cell label hierarchy recovery, leveraging label-aware compressive classification methods and significantly enhancing the accuracy of cell type identification. Then the marker genes are assigned to hierarchical cell labels by their high expressions^25^.

Given the diverse landscape of the above tools, researchers face a challenge to make a good choice, which requires careful consideration of tool performance, compatibility with specific datasets, and suitability for addressing distinct biological questions. The rapid evolution of technology has led to continuous development of new tools and methodologies, further complicating the selection process for the most appropriate tools. Addressing this challenge, the development of a unified platform for benchmarking marker genes should aim to significantly enhance usability while ensuring consistent and comprehensive evaluation metrics for testing various marker gene identification tools. While versatile benchmark projects for scRNA-seq have been conducted in recent years, such as those addressing differential expression analysis^17^, dimension reduction methods^26^, clustering strategies^27^, and data matrix transformations^28^, a benchmark for specialized gene marker identification tools is still absent, let alone a unified platform to perform such assessments in a user-friendly manner.

Therefore, we propose cellMarkerPipe, an adaptable and all-in-one platform designed for cell type-specific marker gene identification and benchmarking. This platform conveniently compiled and wrapped around a list of recent and specialized computational tools for cluster-specific marker gene identification (from 2017 to 2022, see Supplementary Table 1), each contributing to the advancement of marker gene identification in the evolving field of single-cell transcriptomics. Rigorous testing on diverse scRNA-seq datasets from human, mouse, and plant samples (Supplementary Table 2), in conjunction with known markers, validates the robustness of our systematic benchmarking approach. Through a case study, we illustrate the potential applications of the cellMarkerPipe platform in advancing gene therapeutics for targeted cell populations, paving the way for personalized and genomic editing treatments. Implemented in both Python and R, this open-source platform empowers researchers across diverse biological domains with a comprehensive and fully automated protocol for cell-type marker gene identification.

## Results

### Overview of the pipeline

**Figure 1:**
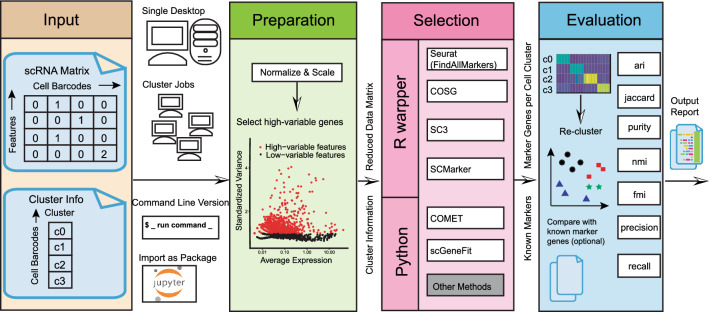
Schematic overview of the pipeline. The cellMarkerPipe comprises three key steps: preparation, selection, and evaluation for marker genes.

The cellMarkerPipe pipeline accepts input in the 10 × format, comprising both a cell-gene matrix and cell cluster labels (see Fig. 1). The output from the pipeline is an evaluation report with comprehensive metrics for the identified marker genes from any selected methodology.

The cellMarkerPipe comprises three core modules: preparation, marker selection, and marker evaluation. Firstly, the preparation step includes normalization, scaling, and potential dimension reduction through the selection of highly variable genes. This step also includes the filtering of low-quality single cells following best practices of Seurat (see Methods). The output of the preparation step is a normalized and scaled cell-gene matrix. Secondly, the marker selection step employs a comma separated two-column file (with cell-barcode and cluster-id) and normalized gene expression data from the previous step to perform gene selection using various methods. The pipeline has already supported multiple R/python environments for the prioritized tools, i.e. Seurat, COSG, SC3, SCMarker, COMET, and scGeneFit, to be compared and benchmarked in this paper. The pipeline also has the capability of allowing researchers to incorporate their own tools or gene selection methods, provided the data formats for cell clusters and normalized expression matrix are compatible with our standards. The output of marker selection is a csv file containing genes specific to each cell type. Thirdly, in the evaluation step, the pipeline assesses the marker genes and outputs a report. The evaluation metrics are based on the re-clustering effect. This means the data is re-clustered based on selected markers to compare the resulting clusters with the cell clusters provided by users and/or calibrated with prior knowledge. Optionally, users can also provide known cluster-specific marker genes to evaluate Precision and Recall scores as additional metrics. Thus, the final evaluation report includes scores such as the Adjusted Rand Index (ARI)^29^, Jaccard index, purity, normalized mutual information (NMI)^30^, and Fowlkes-Mallows Index (FMI) from the re-clustering assessment^31,32^, and precision and recall values for each cell type and overall dataset given known marker genes.

### Systematic benchmarking in diverse testing scenarios

**Figure 2:**
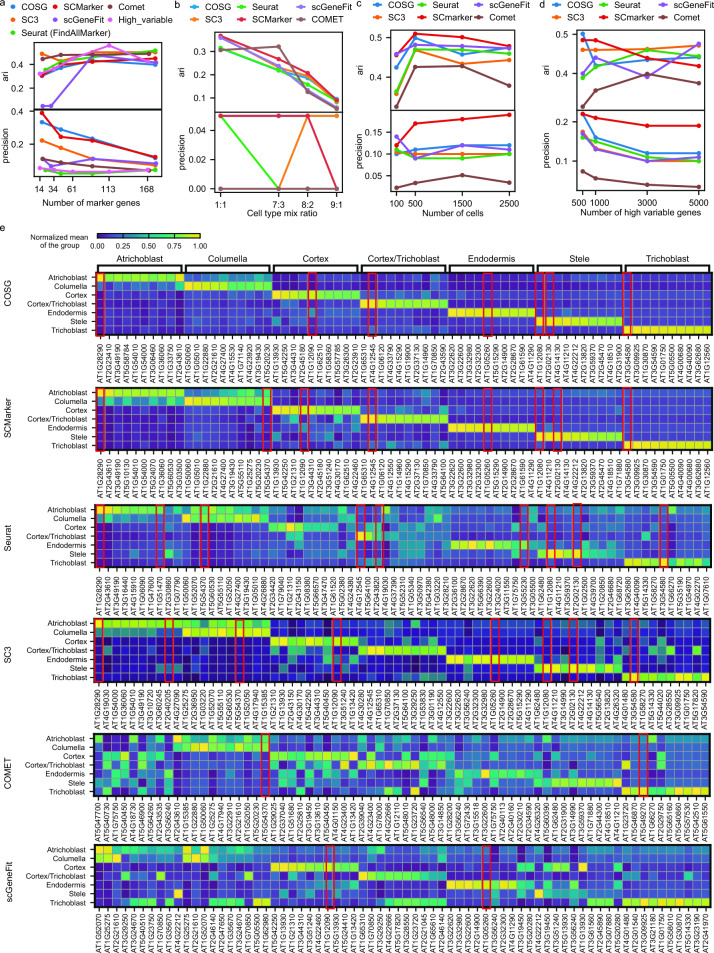
Testing scenarios for various methods. (**a**) Evaluation of methods on Zeisel data with varying numbers of selected marker genes. (**b**) Assessment of methods on Jurkat data with fluctuations in cell type proportions. (**c**) Examination of methods on PBMC data with varying numbers of cells in the input dataset, and (**d**) varying numbers of genes post dimension reduction. (**e**) COSG results showcased for the plant (Arabidopsis root) data, highlighting specific expressions with the top 10 genes in each cluster.

The evaluation of marker genes across different tools raised several critical considerations, including the number of selected marker genes, relative cell type abundance, input cell numbers, and number of highly variable genes at the dimension reduction stage (Supplementary Table 3). These factors that may affect our evaluation have been systematically explored in this section. Among the various efficacy metrics, ARI is frequently used to assess the re-clustering method based solely on the selected marker genes^33^. Additionally, precision is utilized to ascertain true positives among the selected marker genes for each cell type. From ARI and precision curves (Fig. 2a–d), SCMarker and COSG consistently perform well as an overall observation. Meanwhile, other tools exhibit similar performance levels case by case which suggest that they can be satisfactory in various scenarios as well. The complete analytical metrics under various testing cases are all reported and show similar patterns (Supplementary Table 3).

In Case 1 (Fig. 2a), we evaluated the tool performance by controlling the total number of selected marker genes, using a publicly available Zeisel dataset^34^ from the mouse brain. All the methods can be adjusted by certain parameters to reach the same or similar number of selected marker genes. With more marker genes selected, we observed the increasing trend of ARI scores showing an overall improvement in clustering efficacy. As expected, precision instead decreased since the total selected marker genes will dilute the proportion of known marker genes. Among methods, COSG and SCMarker exhibited good precision scores for identifying true positive gene markers at the same or similar number of marker genes being reported. Marker genes identified by COSG and SCMarker also show more specific gene expressions patterns in correspondent cell types from heatmap (Supplementary Fig. 1). Moreover, ARI reaches saturation for most of the methods especially for SCMarker even with fewer than 20 genes (approximately 2 genes in each of the nine cell types). Since all datasets went through dimension reduction by selecting highly variable expressed genes during the preparation step, we selected the similar number of top high variable genes as a control method to indicate the baseline efficacy compared to those specialized tools. This “high variable” method exhibited lower overall precision but comparable ARI score as to other methods at any selected gene numbers, which indicates that the re-clustering on global informative genes may not always offer marker genes in a cell type specific manner. This implies the necessity of comprehensive metrics in our pipeline. Lastly, since the top 10 selected marker genes for each cluster (this is about 90 marker genes in total) in those methods already display a stabilized clustering performance (ARI saturation), we will always report top 10 marker genes for each cluster in later experiments (and in heatmaps) by default.

In Case 2 (Fig. 2b), we tested methods on the relative cell population by Jurkat dataset^35^, an artificial mixture of two distinct cell lines (Jurkat and 293 T). By altering the mixing ratio from 1:1 to an imbalanced scenario up to 9:1 (Jurkat:293 T), we observed a decrease in clustering efficacy when the cell types were more severely imbalanced. However, the effect on precision was not consistently stable since there are only one marker genes in each cell line were considered. Overall, SCMarker exhibited relatively higher re-clustering effectiveness in this test of imbalanced cell types, with several other tools demonstrating similar or comparable performance. From the heatmap visualization of marker gene expression specificity, SCMarker, COSG, Seurat and SC3 all display strong patterns (Supplementary Fig. 2).

In Case 3 (Fig. 2c), regarding experimental throughput, we varied the total number of input cells from 100 to 2500 using PBMC-10 K dataset^35^ to investigate the impact for marker genes identification. We observed an enhanced clustering effectiveness (ARI) and precision score with over 500 cells, equating to roughly 50 cells per cluster. Generally, more cells can provide better distribution of gene expressions but may also bring in more noise. The gene expression specificity pattern is not very observable when using 2500 cells and 5000 genes as inputs in all methods (Supplementary Fig. 3). Given current technological capabilities which enable the processing of over 5000 cells, the limitation of the input cell number is of minor consideration, except for rare cell types.

In Case 4 (Fig. 2d), we tested the effect of input gene numbers (highly variable genes from top 500 to 5,000 in dimension reduction) using PBMC-10 K dataset^35^. This experiment illustrated that input gene numbers (often after dimension reduction with highly variable genes) may not significantly affect clustering efficacy but do impact precision. SCMarkers and COSG displayed relatively good precision scores in this testing, but their gain in clustering accuracy is minor given all tools including Seurat and SC3 indicates comparable re-clustering scores. This result also emphasizes that leveraging a substantial number of highly variable genes is beneficial for enhancing clustering efficacy^36^ but may not necessarily serve as specific markers for cell types. This underscores that genes playing crucial roles in overall clustering performance are not necessarily cell type-specific marker genes.

In Case 5 (Fig. 2e), we utilized a plant dataset derived from Arabidopsis root single cells^37^ to visualize the standardized gene expression specificity using the top 10 selected gene markers in each cell type. Heatmaps can visually represent the marker genes that influenced these cell clusters^38^. SCMarker, SC3 and COSG played important roles in identifying type-specific expressed genes, with the yellow colors indicating higher specificity of cell type expressions. Additionally, the red box highlights the re-discovery of known marker genes. The marker genes selected by SCMarker, SC3, Seurat and COSG included more reported known marker genes (in red boxes) than those selected by other methods affirming the superior performance of SCMarker, SC3, Seurat and COSG in this context.

### Comparative studies in human and mice gut tissues

**Figure 3:**
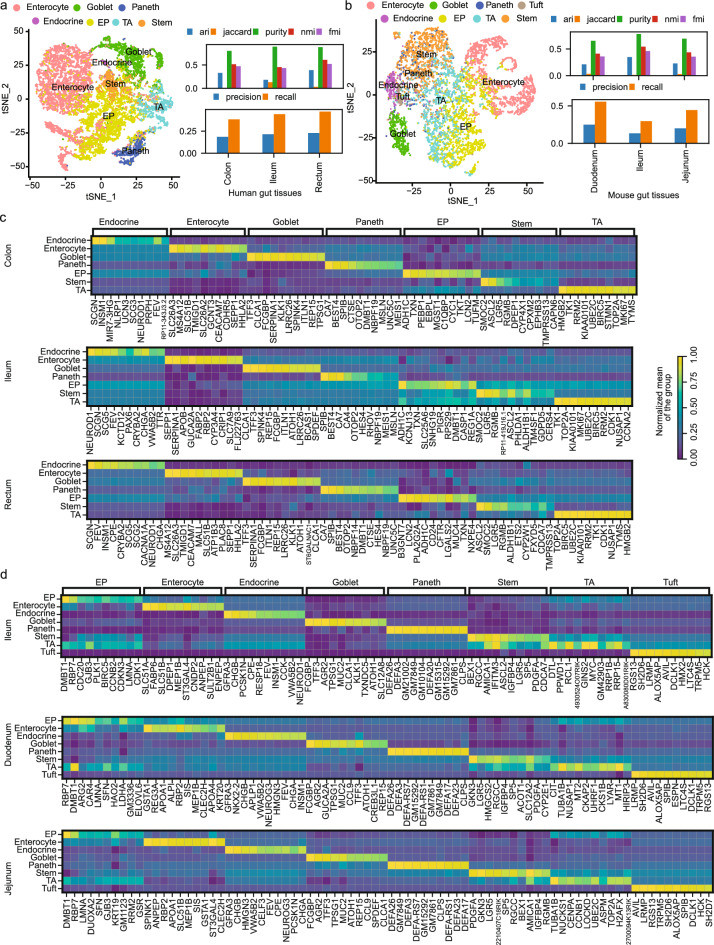
Performance of COSG on single-cell data. (**a**) Evaluation of COSG performance on human gut single-cell data. (**b**) Evaluation of COSG performance on mice gut single-cell data. (**c**) Top 10 gene expressions per cell type identified by COSG for human gut samples. (**d**) Top 10 gene expressions per cell type identified by COSG for mice gut samples.

In this experiment, we utilized datasets from both human and mice gut cells^39,40^, to conduct comprehensive comparisons across our selected methodologies. Initially, we reconstructed and displayed single-cell clusters colored on the given cell type labels identified in the respective studies (Fig. 3a,b). In the human colon, ileum, and rectum tissues, we obtained marker genes for cell types of Paneth, Goblet, Enterocyte, Stem-cell, Enteroendocrine, EP (Enterocyte progenitor), and TA (transit-amplifying), while in mouse duodenum, ileum, and jejunum tissues, we obtained marker genes for Enterocyte, Tuft, Goblet, Enteroendocrine, Stem-cell, TA, EP, and Paneth cell types. Overall, COSG achieved consistently better gene recall scores in human ileum rectum and mouse duodenum, while SCMarker, SC3 and Seurat performed better in other tissues (Supplementary Table 4). The performance patterns of the computational method COSG remained relatively consistent across both human and mouse, as well as across various tissue types (Fig. 3a,b). From heatmaps, the specific gene expression values within each cell type were distinctly elucidated by the top 10 marker genes identified through the COSG method (Fig. 3c,d). Genes selected by SCMarker also displayed specific cell type expression patterns (Supplementary 4–9) across human and mice tissues. While we did observe shared markers, such as TFF3, ATOH1, and FCGBP in Goblet cells and LGR5 in Stem-cells from both human and mouse ileum data, it is important to notice that, overall, the marker genes identified in human and mice datasets exhibited differences (Fig. 3c,d).

### Running time comparison for various methods

**Figure 4:**
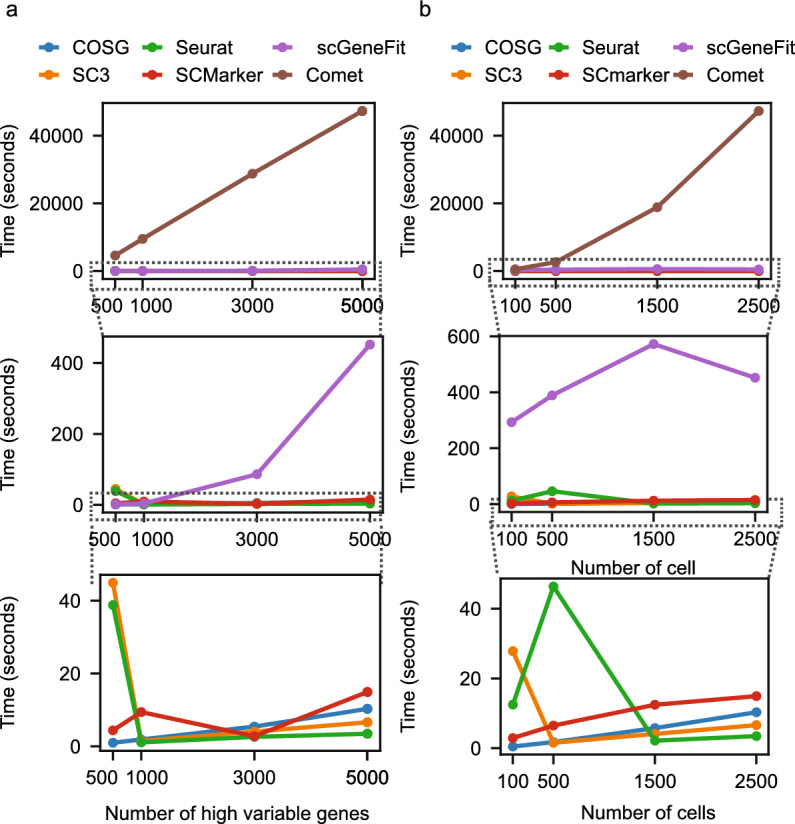
Running time measurement in seconds for varied input parameters. (**a**) Variation in running time with changes in the number of genes. (**b**) Variation in running time with changes in the number of cells in the input data. These computational tasks were executed on a computing node with 10G of allocated memory, running a single process on standard CPUs at the Holland Computing Center, University of Nebraska Lincoln.

In this experiment, we conducted a thorough analysis of the running time complexity and scalability across various methods using the PBMC dataset. By manipulating the number of genes and cells as input variables, we meticulously measured the running time taken for the marker gene selection step in seconds across different tools (Fig. 4a,b). COMET exhibited challenges in scalability as it necessitates the examination of marker gene combinations, making it less efficient when faced with an increased number of genes or cells. Similarly, scGeneFit displayed relatively extended processing times due to the evaluation of gene networks based on positive and negative correlations. In contrast, the running time for the remaining tools demonstrated similar performance, showing little variation in response to changes in input variables.

### Examination of cell makers and cell types in gene therapy

**Figure 5:**
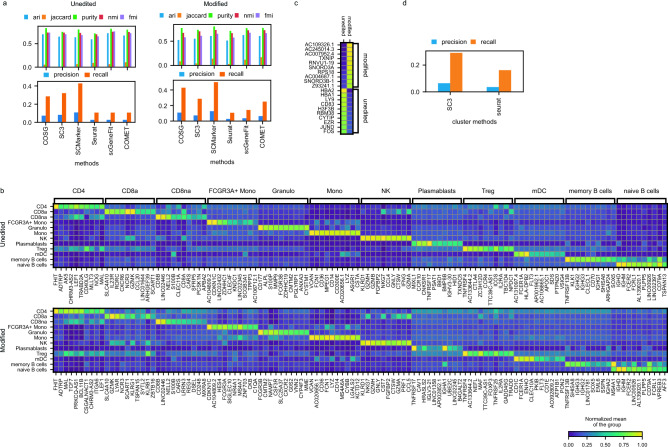
Comparative performance in blood samples. (**a**) Performance of methods in unedited and modified blood samples from the same patient. (**b**) Top 10 marker gene expressions per cell type identified by COSG for unedited and modified blood samples. (**c**) Top 10 gene expressions for B cells. (**d**) Influence of clustering method on recall and precision.

A recent clinical trial^41^ explores gene editing-based therapy for children with transfusion-dependent β-thalassemia caused by HBB gene mutation. By targeting the BCL11A enhancer, researchers aim to induce γ-globin expression to compensate the globin deficiency. Two children received edited stem cells, achieving successful engraftment and transfusion independence for over 18 months. Single cell data from this study provide exploratory analysis, which revealed no notable side effects. Here we used our cellMarkerPipe to re-evaluate the single cell data from this medical research from one of the children with both unedited and modified blood cells using six methods. We obtained the cell clusters and gene markers from the original publication. With the known blood cell markers^41^, SCMarker, SC3 and COSG identified a good set of marker genes (Fig. 5a) according to their precision and recall scores. For the clustering effectiveness, all tools displayed comparable scores with their maker genes.

The COSG selected top 10 marker genes were visually represented for each cell type, showcasing their specific expressions in a heatmap (Fig. 5b). This pattern can be also observed in SCMarker identified marker genes, but not observed clearly from other tools (Supplementary Fig. 10–11). Most of these markers were shared across unedited and modified samples in identified blood cell types. Interestingly, when combining B cells (naïve and memory) in these two samples (Fig. 5c), BCL11a did not emerge as a distinguishing marker between edited and unedited cases. This overall analysis suggests that the gene editing effect may not significantly impact major mature cell types according to the marker gene selections. Additionally, we explored potential uncertainties arising from different clustering methods, such as SC3 (Fig. 5d). SC3 and Seurat were both controlled to generate twelve clusters, and we obtained top ten marker genes for each cluster by COSG. While minor differences were observed in overall precision and recall for known cell markers, it's important to notice that the choice of clustering method in scRNA-seq analysis may influence marker gene selection and downstream analysis. Nonetheless, this variation, given commonly used cell clustering methods (such as Seurat is used by default in our cellMarkerPipe), shall not pose a major concern for a real-world data analysis.

## Discussion

CellMarkerPipe places a strong emphasis on utilizing cell cluster aware marker gene identification methods. While general dimension reduction or feature selection methods hold their own advantages in specific contexts, the prioritization of specialized marker gene selection techniques in this platform ensures assessment of marker gene quality and specificity that can emulate the cell sorting approach.

CellMarkerPipe offers a comprehensive range of metrics, including clustering effectiveness scores and precision/recall values based on known markers. The distinguishing metrics that determine one tool's superiority over another may vary depending on the conditions and datasets. In our testing, we found that metrics were often similar among tools, though differences could arise. While our conclusions may sometimes favor specific tools, cellMarkerPipe presents all metrics to users, providing them with sufficient information for analysis and judgment.

Among the tools integrated within cellMarkerPipe, SCMarker and COSG both exhibit commendable stability and consistency in their performance. Particularly, COSG stands out for its adeptness and efficiency in employing simple cosine distance measures. This characteristic lends COSG a high degree of reliability in marker gene identification across diverse datasets, underscoring its suitability for robust single-cell analyses.

The unique feature of COMET, its ability to identify combinatorial gene markers, has not been extensively tested and fairly benchmarked in this research. Indeed, the gene panel identification of COMET represents a distinctive advantage, revealing intricate relationships among genes and offering significant biological insights into complex regulatory networks. Nevertheless, this unique capability of COMET comes with the trade-off of expensive time complexity.

Seurat, as a widely adopted tool in single-cell analysis, provides valuable utility through its FindAllMarkers function. It's crucial to recognize that while Seurat is convenient to use and should be suffice for diverse applications, it might not always be the optimal choice for the comprehensive discovery of marker genes. This consideration implies the importance of evaluating other marker identification approaches.

We limit our tests by default settings for differential expression (DE) gene statistical methods in two widely used packages, Seurat and SC3, recognizing the diverse options and settings explored in other DE benchmark projects^17,18^. When utilizing differential expression (DE) statistical methods, as they may select top-ranked DE genes that are highly expressed in target cells and a small group of nontarget cells, potentially leading to erroneous identification as marker genes^22^. This highlights the importance of incorporating other more sophisticated marker gene identification methods besides the DE approaches.

Overall, CellMarkerPipe stands out as a new, standardized and uniform platform designed for the identification of cell-specific marker genes, coupled with comprehensive benchmarking capabilities. In practical applications, researchers have the flexibility to seamlessly integrate cellMarkerPipe with any gene selection tool as a plugin, enabling them to efficiently pinpoint marker genes tailored to their specific research objectives. Furthermore, users can readily access an informative evaluation report, thereby ensuring a thorough assessment of the identified markers. This streamlined process exemplifies the adaptability and user-centric nature of cellMarkerPipe, providing a valuable resource for researchers seeking precise and reliable marker gene identification in their single-cell transcriptome analyses.

## Methods

### Pipeline development

The cellMarkerPipe was developed by both python and R environment. For the simplicity and compatibility concerns, each of the methods we selected has its own working environment in the development of the pipeline. The installation of working environment correspondent to each of the tools is listed in the github (see code availability section). The pipeline was implemented in both command line version and the python modules. Python modular functions are tested in Jupyternotebooks in both a single computer and a computer cluster.

The preprocessing and data preparation step involves filtering data based on criteria such as the minimum and maximum gene count per cell and the percentage of mitochondrial genes per cell, following a widely adopted protocol in Seurat. For re-clustering evaluation, Seurat was employed, as it is the most prevalent package for cell clustering. In the final experiment using a clinical dataset, we also implemented SC3 clustering to compare its clustering effectiveness in the preparation step. The same data formats were prepared from SC3 clustering output so that the marker selection step and evaluation step were run without any revision in procedures.

### Benchmark from datasets and tools

The datasets in this study were chosen from popular public scRNA-seq datasets widely used in cell clustering and annotations. These datasets should have a clear demonstration of known cell types and marker genes being used in their study. We also aimed to maximize the species and tissue coverage with diverse application scenarios by these datasets. The data matrices and cell type information were downloaded from NCBI or their publication repository (Supplementary Table 2). The ground truth for the marker genes from each of the datasets were collected from the original publication or relevant studies they refer to (Supplementary Table 5). To make sure the fair comparison of the total gene selections in different tools, we ensure the total number of selected genes are comparable based on the adjustment of the parameters in each tool (in experiments mentioned in Fig. 2a), or simply report the top ten marker genes per cluster (in experiments other than Fig. 2a). For the method scGeneFit, since it’s hard to directly control the selected gene numbers, we tuned the other parameters to make sure the number of selected genes is closest to the testing cases of all other methods. The values of parameters we used in this study for all tools ensure the proper comparison and reproducibility of this benchmark and used as default settings in our pipeline (Supplementary Table 6). When reporting the total selected marker genes (Supplementary Table 7) from each dataset, since some clusters may share the marker genes, the non-redundant numbers were reported in the paper and evaluated in ARI and precision scores. The marker genes for individual cell types were also reported (Supplementary Table 7) and used in gene expression heatmaps (Supplementary Figs. 1–11).

The heatmaps showing the cell type specific gene expressions for selected marker genes per group were generated by Scanpy sc.pl.matrixplot function^42^ at “standard_scale” mode. This mode is to standardize the given gene expressions into the values between 0 and 1, meaning for each variable or group the values subtract the minimum and divide each by its maximum.

Detailed statistics for these various experiments and more heatmaps were shown in Supplementary Table 3,4 and Supplementary Figs. 1–11.

### Evaluation scores in benchmark reports

The final evaluation report provides scores for metrics are either from re-clustering by Seurat default or comparison of the known gene markers. The re-clustering based metrics are Adjusted Rand Index (ARI), Jaccard index, purity, normalized mutual information (NMI), Fowlkes-Mallows Index (FMI). The comparisons of the known markers provide precision and recall. These metrics are demonstrated in the following formula. These scores are obtained by scikit-learn package^43^.

ARI is a measure of consistency between the observation and expected cluster results. The observation clusters *C*^*o*^ is based on identified marker genes, while the expected clusters *C*^*E*^ is based on the true cell cluster labels. Assume n is the total number of cells. The number *n*_*ij*_ represents the cell numbers in both i-th cluster in observation *C*^*o*^ and j-th cluster in expectation *C*^*E*^. The number *n*_*i.*_ represents the cell numbers in i-th cluster in observation *C*^*o*^, and the number *n*_*.j*_ represents the cell numbers in j-th cluster in expectation *C*^*o*^. The ARI can be calculated by the formula below. The ARI value will be in the range [0, 1]. Higher values indicate better agreement between the predicted (observed) and true (expected) clusters by the identified gene markers.

1 $$ARI\left( {C^{o} ,C^{E} } \right) = \frac{{{{\mathop \sum \nolimits_{i,j} \left( {\begin{array}{*{20}c} {n_{ij} } \\ 2 \\ \end{array} } \right) - \left[ {\mathop \sum \nolimits_{i} \left( {\begin{array}{*{20}c} {n_{i.} } \\ 2 \\ \end{array} } \right)\mathop \sum \nolimits_{j} \left( {\begin{array}{*{20}c} {n_{.j} } \\ 2 \\ \end{array} } \right)} \right]} \mathord{\left/ {\vphantom {{\mathop \sum \nolimits_{i,j} \left( {\begin{array}{*{20}c} {n_{ij} } \\ 2 \\ \end{array} } \right) - \left[ {\mathop \sum \nolimits_{i} \left( {\begin{array}{*{20}c} {n_{i.} } \\ 2 \\ \end{array} } \right)\mathop \sum \nolimits_{j} \left( {\begin{array}{*{20}c} {n_{.j} } \\ 2 \\ \end{array} } \right)} \right]} {\left( {\begin{array}{*{20}c} n \\ 2 \\ \end{array} } \right)}}} \right. \kern-0pt} {\left( {\begin{array}{*{20}c} n \\ 2 \\ \end{array} } \right)}}}}{{{{\frac{1}{2}\left[ {\mathop \sum \nolimits_{i} \left( {\begin{array}{*{20}c} {n_{i.} } \\ 2 \\ \end{array} } \right) + \mathop \sum \nolimits_{j} \left( {\begin{array}{*{20}c} {n_{.j} } \\ 2 \\ \end{array} } \right)\left] - \right[\mathop \sum \nolimits_{i} \left( {\begin{array}{*{20}c} {n_{i.} } \\ 2 \\ \end{array} } \right)\mathop \sum \nolimits_{j} \left( {\begin{array}{*{20}c} {n_{.j} } \\ 2 \\ \end{array} } \right)} \right]} \mathord{\left/ {\vphantom {{\frac{1}{2}\left[ {\mathop \sum \nolimits_{i} \left( {\begin{array}{*{20}c} {n_{i.} } \\ 2 \\ \end{array} } \right) + \mathop \sum \nolimits_{j} \left( {\begin{array}{*{20}c} {n_{.j} } \\ 2 \\ \end{array} } \right)\left] - \right[\mathop \sum \nolimits_{i} \left( {\begin{array}{*{20}c} {n_{i.} } \\ 2 \\ \end{array} } \right)\mathop \sum \nolimits_{j} \left( {\begin{array}{*{20}c} {n_{.j} } \\ 2 \\ \end{array} } \right)} \right]} {\left( {\begin{array}{*{20}c} n \\ 2 \\ \end{array} } \right)}}} \right. \kern-0pt} {\left( {\begin{array}{*{20}c} n \\ 2 \\ \end{array} } \right)}}}}$$

The Jaccard Index measures the similarity between two sets by comparing the intersection (common elements in clusters) with the union (total elements in clusters) of the sets, often used to assess the similarity of clustering results^44^. The calculation is as follows given the i-th cluster in observation *C*^*o*^ and j-th cluster in expectation *C*^*E*^. The overall Jaccard Index is based on the mean of all the cluster-wise comparisons.

2 $$JI\left( {C_{i}^{o} , C_{j}^{E} } \right) = \frac{{\left| {C_{i}^{o} \cap C_{j}^{E} } \right|}}{{\left| {C_{i}^{o} \cup C_{j}^{E} } \right|}} = \frac{{n_{ij} }}{{n_{i.} + n_{.j} - n_{ij} }}$$

NMI quantifies the mutual dependence between two sets of labels, adjusted for chance, providing a measure of the agreement between the two clustering results. In detail, NMI is mutual information between observation and expectation *I*(*C*^*o*^, *C*^*E*^) normalized by entropies of each *H*(*C*^*o*^) and *H*(*C*^*E*^). NMI can be calculated by the following formula.

3 $$NMI\left( {C^{o} ,C^{E} } \right) = \frac{{I\left( {C^{o} ,C^{E} } \right)}}{{\sqrt {H\left( {C^{o} } \right) H\left( {C^{E} } \right) } }} = \frac{{\mathop \sum \nolimits_{i,j} n_{ij} \log \left( {\frac{{n*n_{ij} }}{{n_{i.} *n_{.j} }}} \right)}}{{\sqrt {\mathop \sum \nolimits_{i} n_{i.} \log \left( {\frac{{n_{i.} }}{n}} \right)*\mathop \sum \nolimits_{j} n_{.j} \log \left( {\frac{{n_{.j} }}{n}} \right)} }}$$

FMI assesses the similarity between two cluster results by computing the geometric mean of the pairwise precision and recall. TP (true positive), FP (false positive) and FN (false negative) are based on the cell label contingency table between i-th cluster in observation *C*^*o*^ and j-th cluster in expectation *C*^*E*^.

4 $$FMI\left( {C_{i}^{o} , C_{j}^{E} } \right) = \sqrt {\frac{TP}{{TP + FP}}*\frac{TP}{{TP + FN}}}$$

Besides the above re-clustering metrics based on the identified marker genes, if user provides the “true” or expected marker gene sets, our pipeline also calculates the Precision and Recall scores to show whether the predicted marker genes are consistent with biological validated or “true” marker gene sets. Precision means the percentage of the corrected predicted genes among all predicted genes, while Recall means the percentage of the corrected predicted genes among all expected true results. Our pipeline will report cluster-wise Precision and Recall scores for each cluster, as well as the overall Precision and Recall scores when the marker genes from all clusters are pooled together. Figures and tables in this paper only display the overall Precision and Recall scores for easy comparison among methods. Overall, cellMarkerPipe provides very comprehensive metrics in the final evaluation report.

### Supplementary Information

Supplementary Table S1.

Supplementary Table S2.

Supplementary Table S3.

Supplementary Table S4.

Supplementary Table S5.

Supplementary Table S6.

Supplementary Table S7.

Supplementary Figures.

## Data Availability

Publicly available scRNA-seq datasets used in this study are listed in Supplementary Table 2. The resulted scores and complete statistics are reported in Supplementary Table 3 and 4. All identified marker genes from this study are available in Supplementary Table 7.

## Code availability

Source code for cellMarkerPipe is available in github repository (https://github.com/yao-laboratory/cellMarkerPipe). CellMarkerPipe can be run in both command line version and jupyternotebook version. Testing dataset and example codes are all available in this github repository.
