# Supplementary material for: CellMarkerPipe: cell marker identification and evaluation pipeline in single cell transcriptomes
Source: Sci Rep. 2024 Jun 7;14:13151. doi: 10.1038/s41598-024-63492-z (PMC11161599; doi:10.1038/s41598-024-63492-z)

cellMarkerPipe: Cell Marker Identification and Evaluation Pipeline in Single Cell Transcriptomes

Supplementary Figures

Supplementary Figure 1. Heatmap for Zeisel data.

Supplementary Figure 2. Heatmap for Jurkat data at 50:50 mixture ratio.

Supplementary Figure 3. Heatmap for PBMC data with input of 2500 cells and 5000 highly variable genes.

Supplementary Figure 4. Heatmap for human ileum data.

Supplementary Figure 5. Heatmap for human rectum data.

Supplementary Figure 6. Heatmap for human colon data.

Supplementary Figure 7. Heatmap for mouse ileum data.

Supplementary Figure 8. Heatmap for mouse duodenum data.

Supplementary Figure 9. Heatmap for mouse jejunum data.

Supplementary Figure 10. Heatmap for patient unedited blood sample.

Supplementary Figure 11. Heatmap for patient blood sample after BCL11A enhancer modification.

Supplementary Figure 1

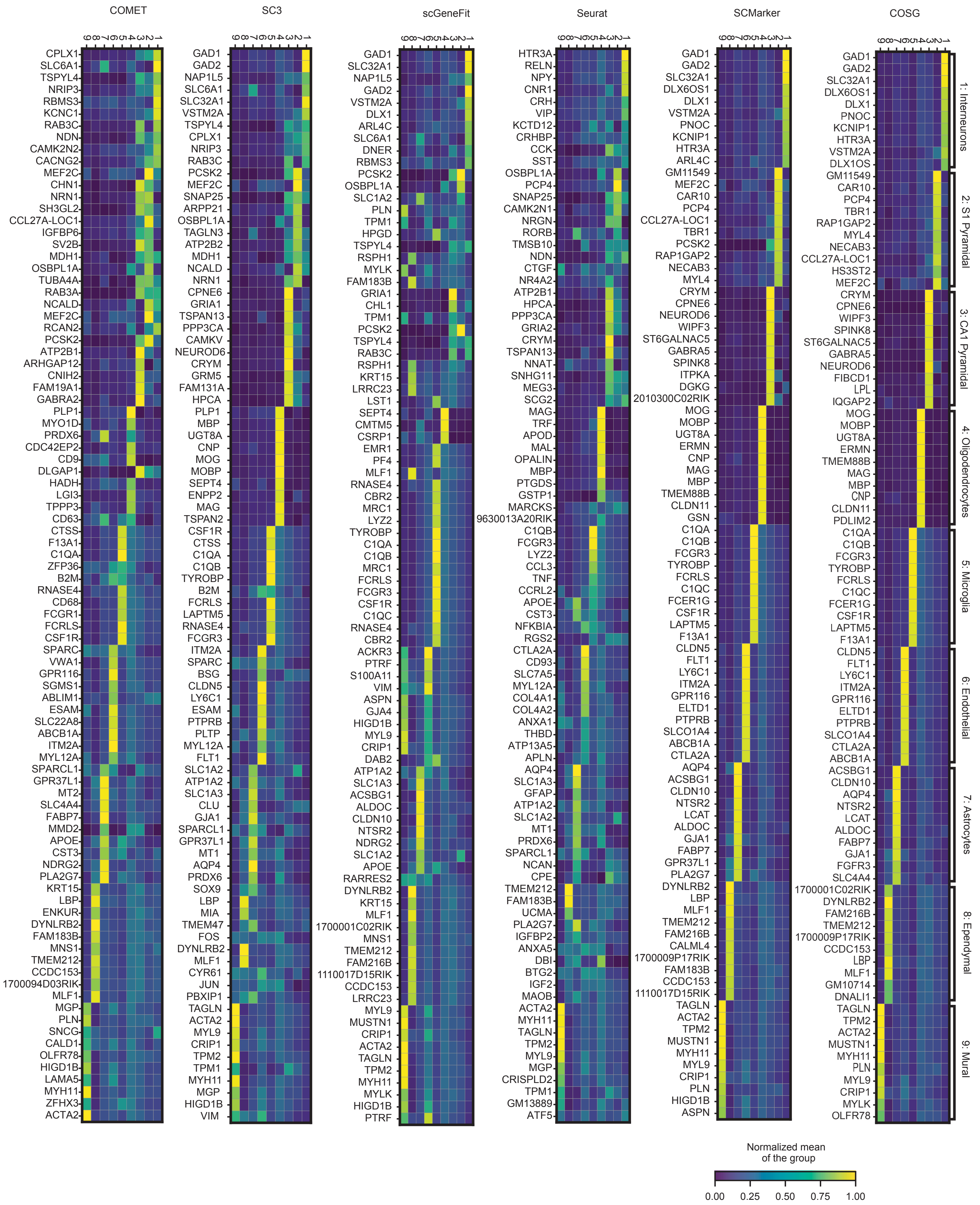

Supplementary Figure 2

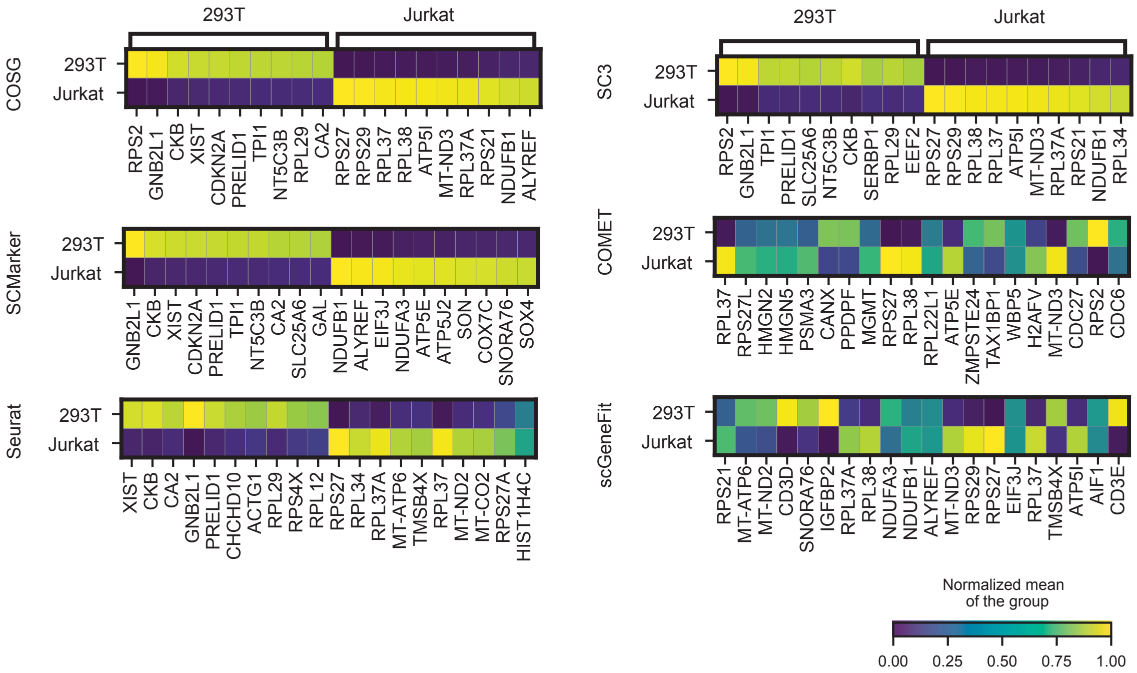

Supplementary Figure 3

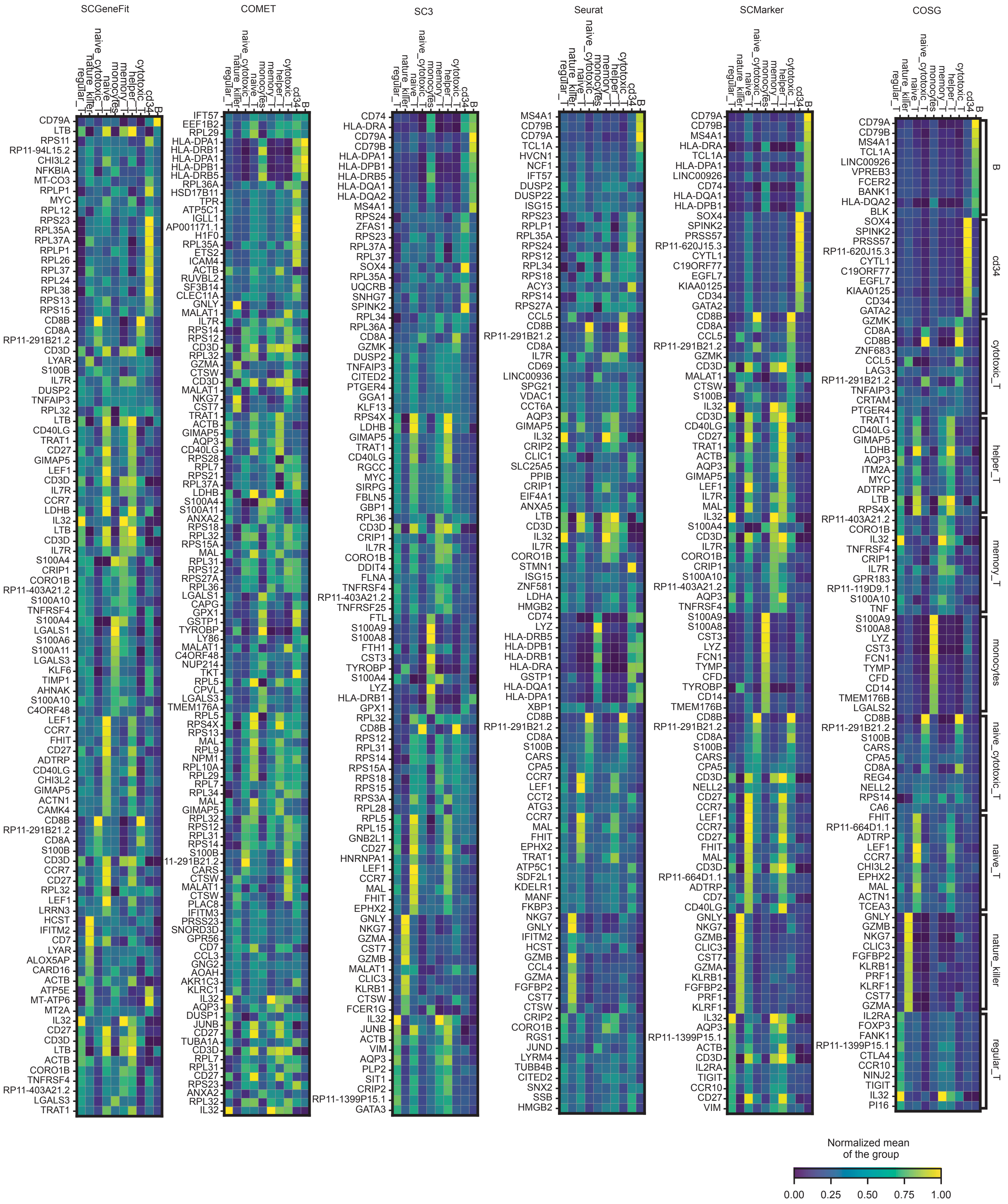

Supplementary Figure 4

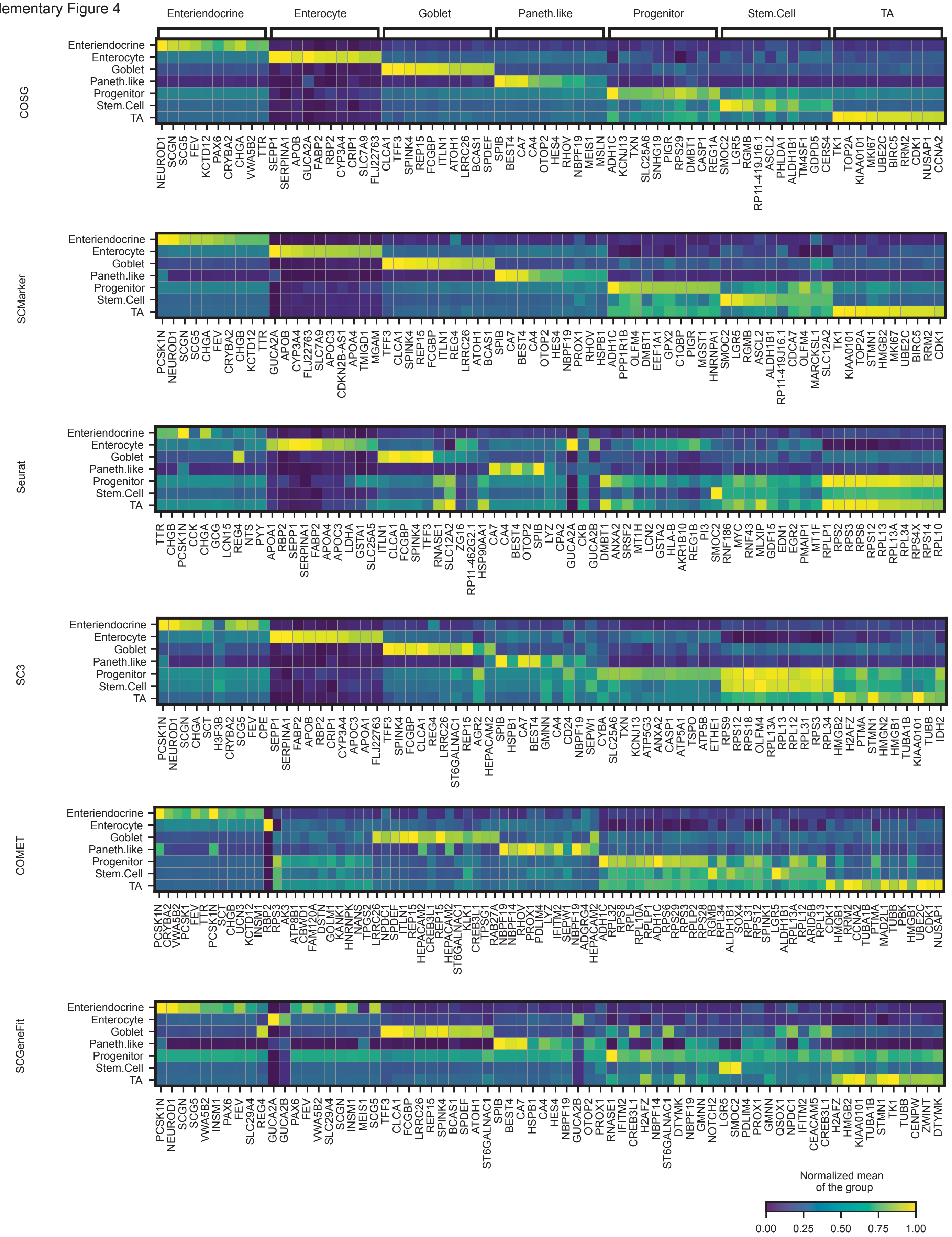

Supplementary Figure 5

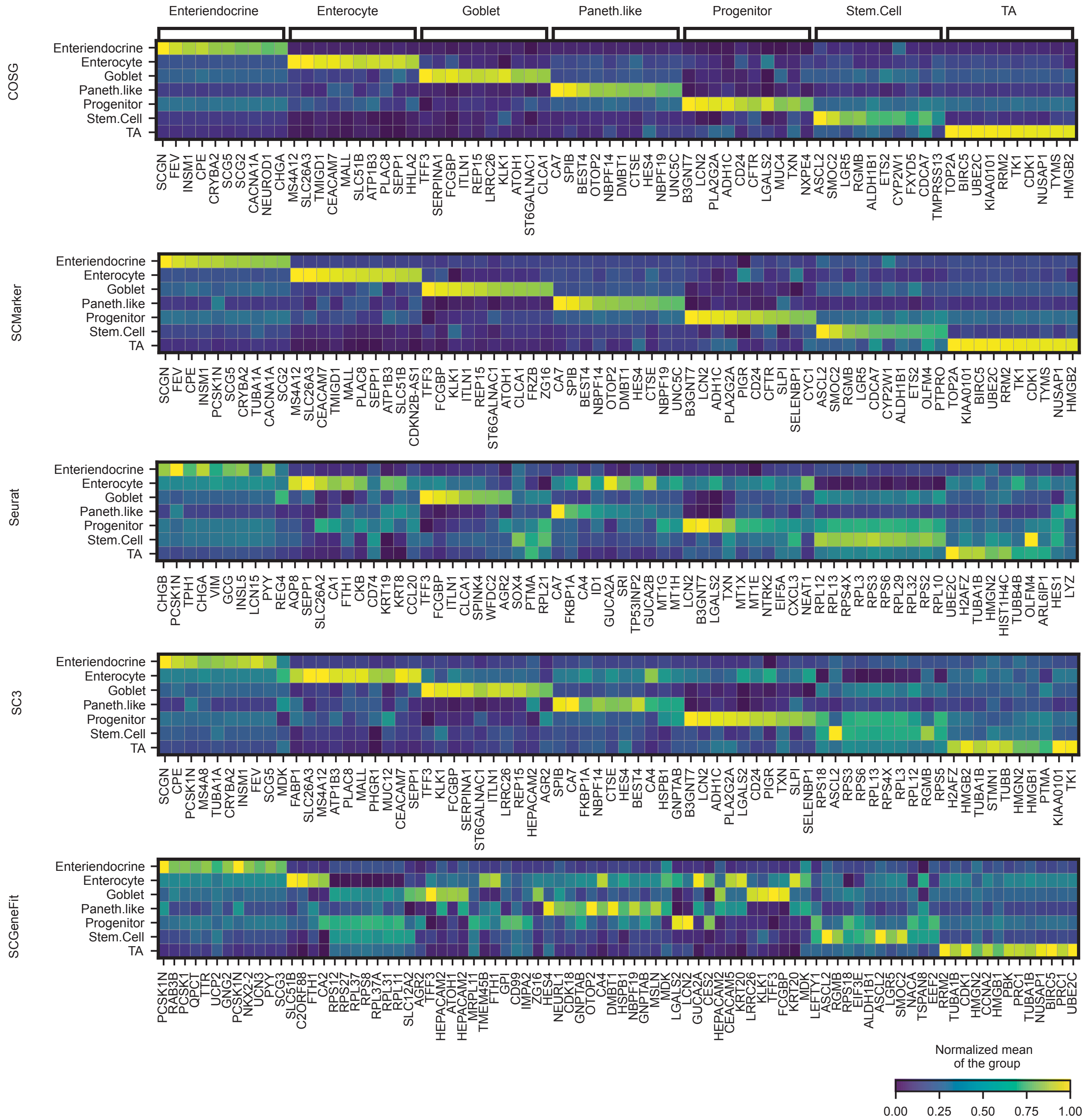

Supplementary Figure 6

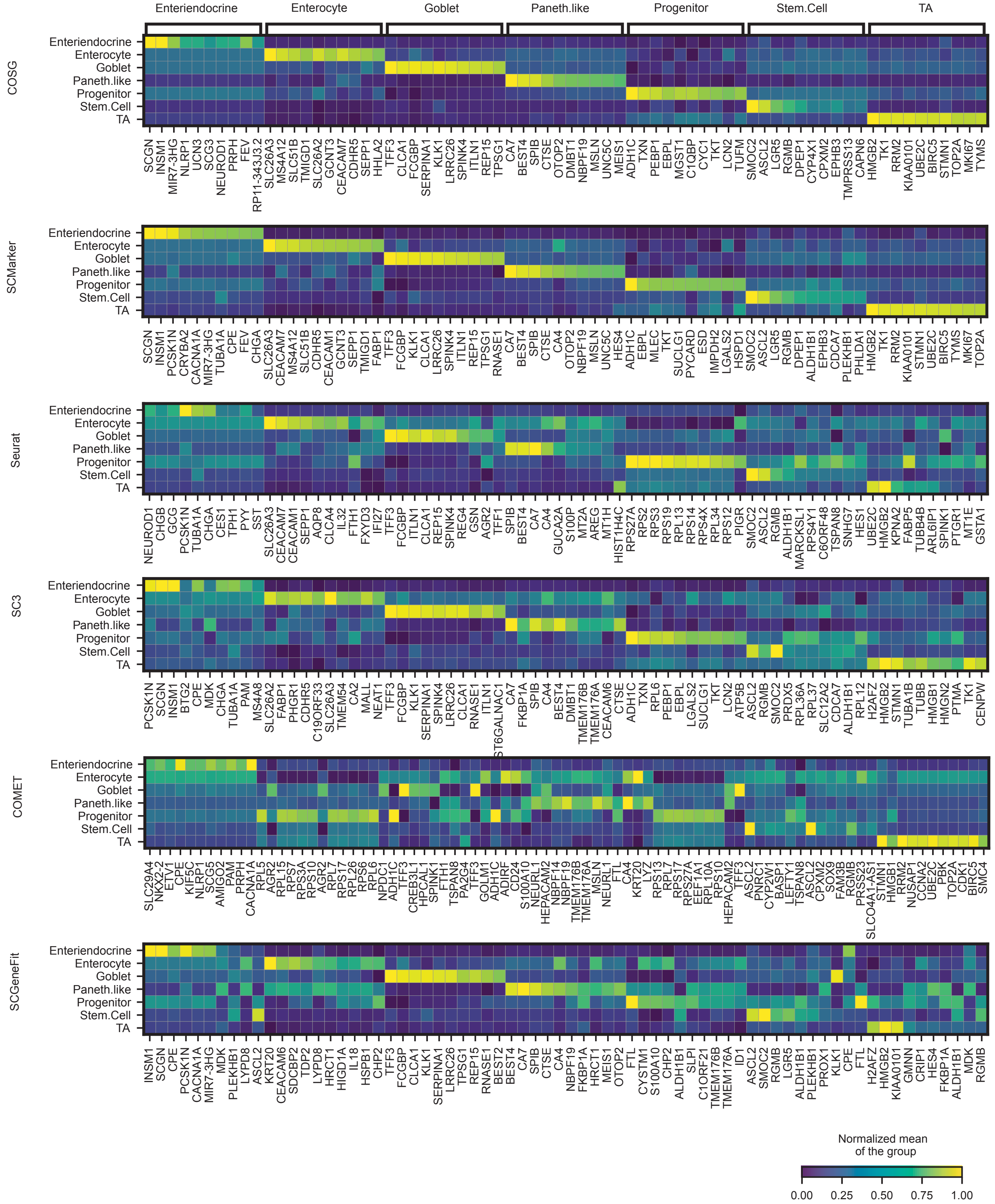

Supplementary Figure 7

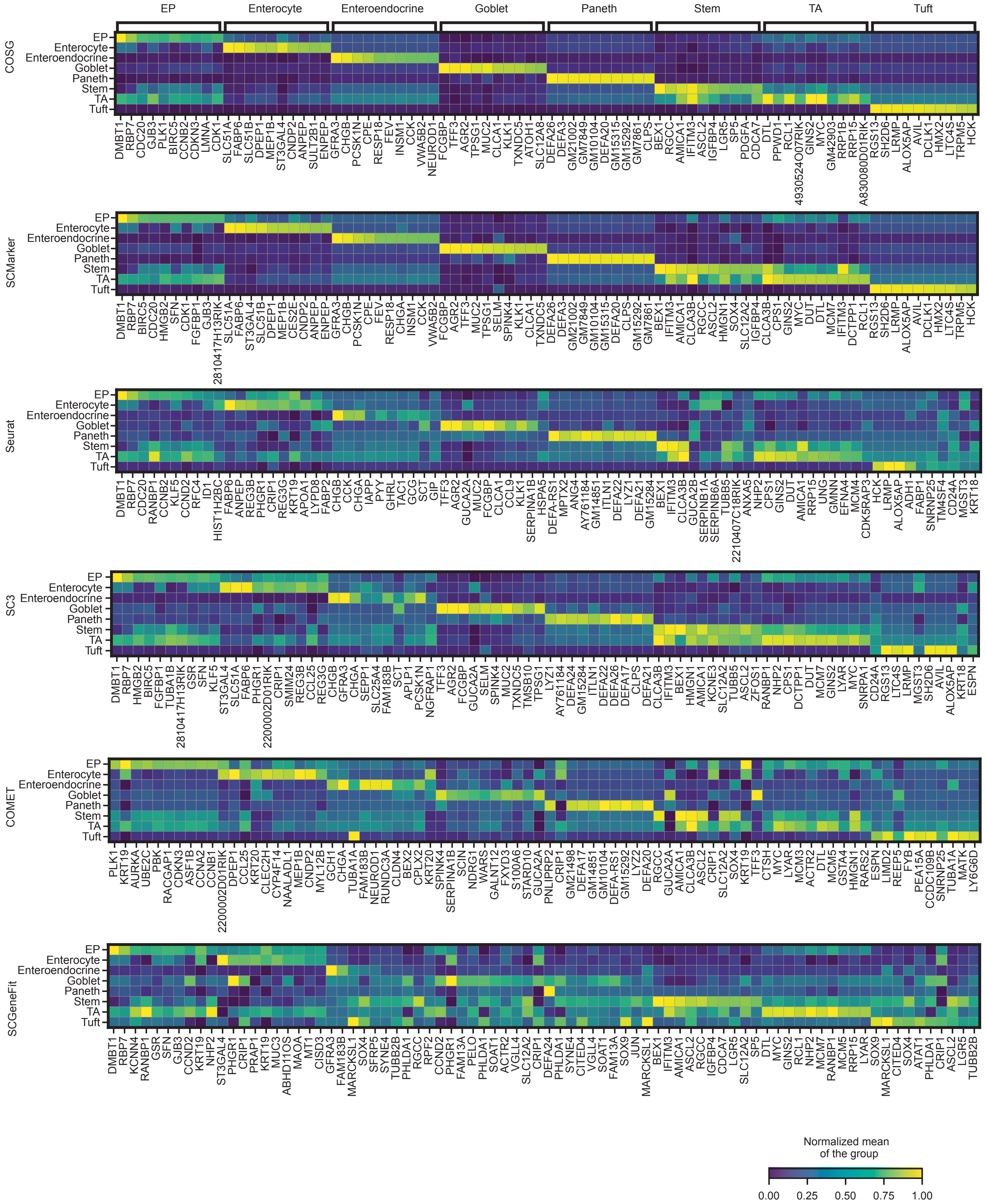

Supplementary Figure 8

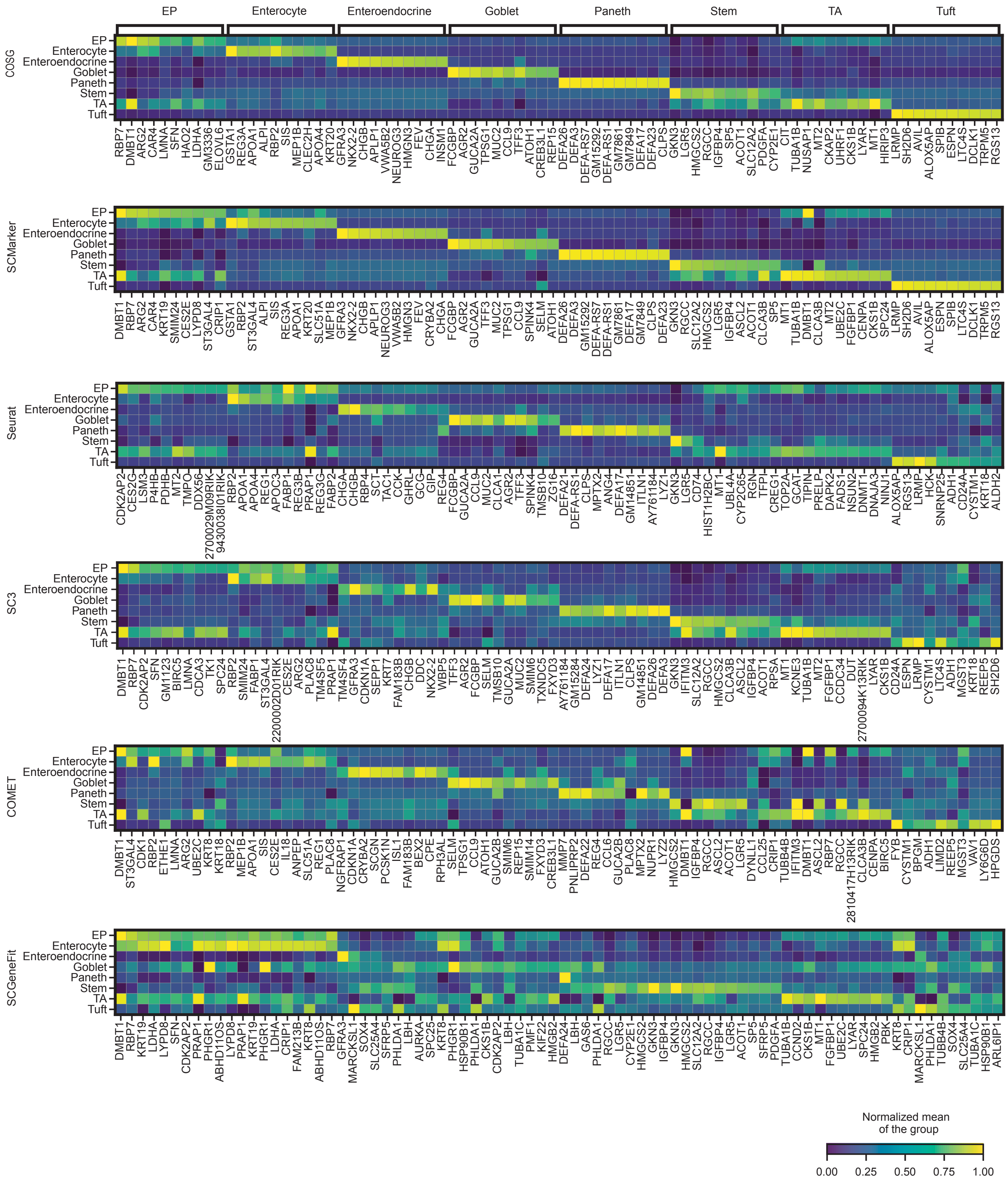

Supplementary Figure 9

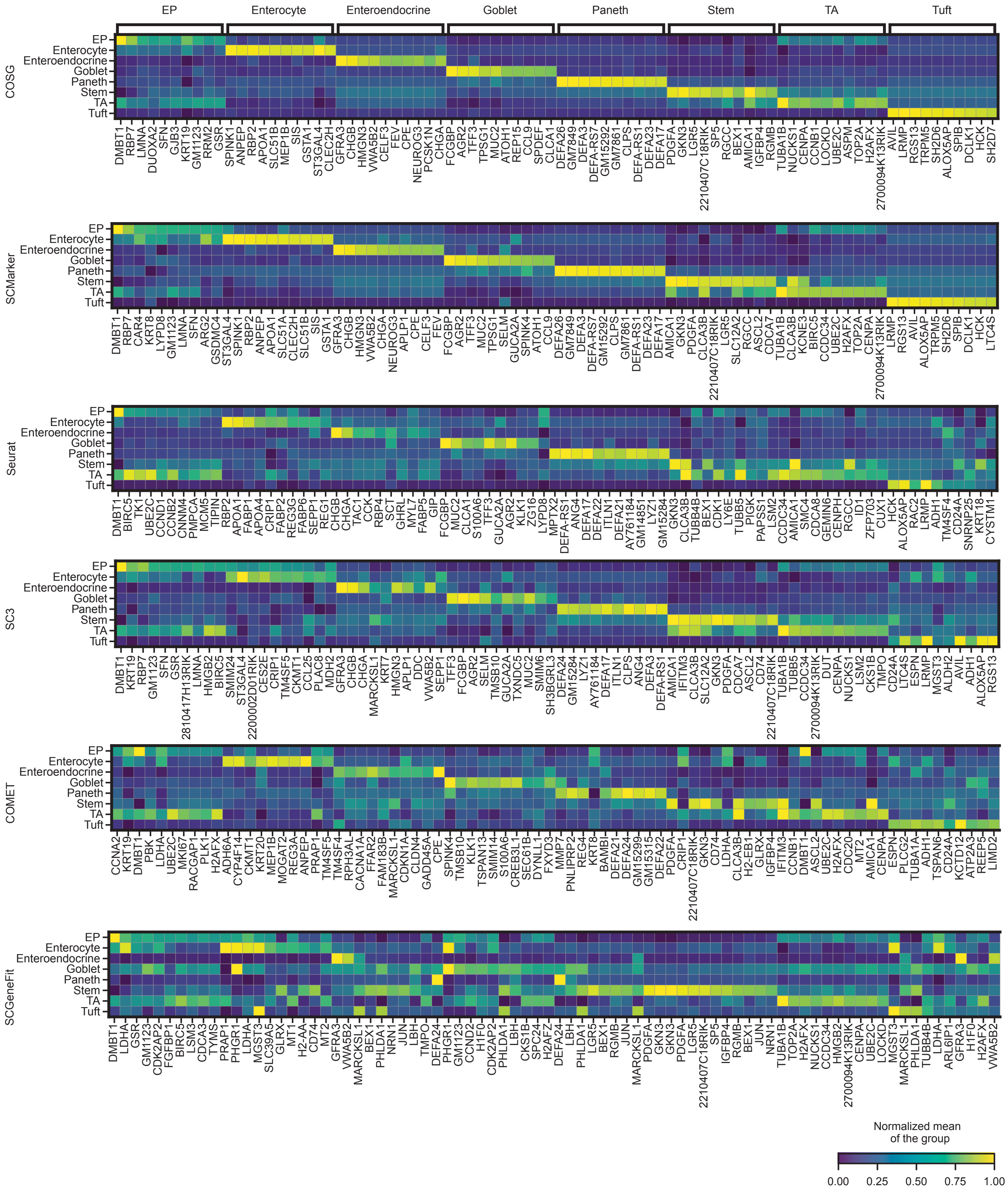

Supplementary Figure 10

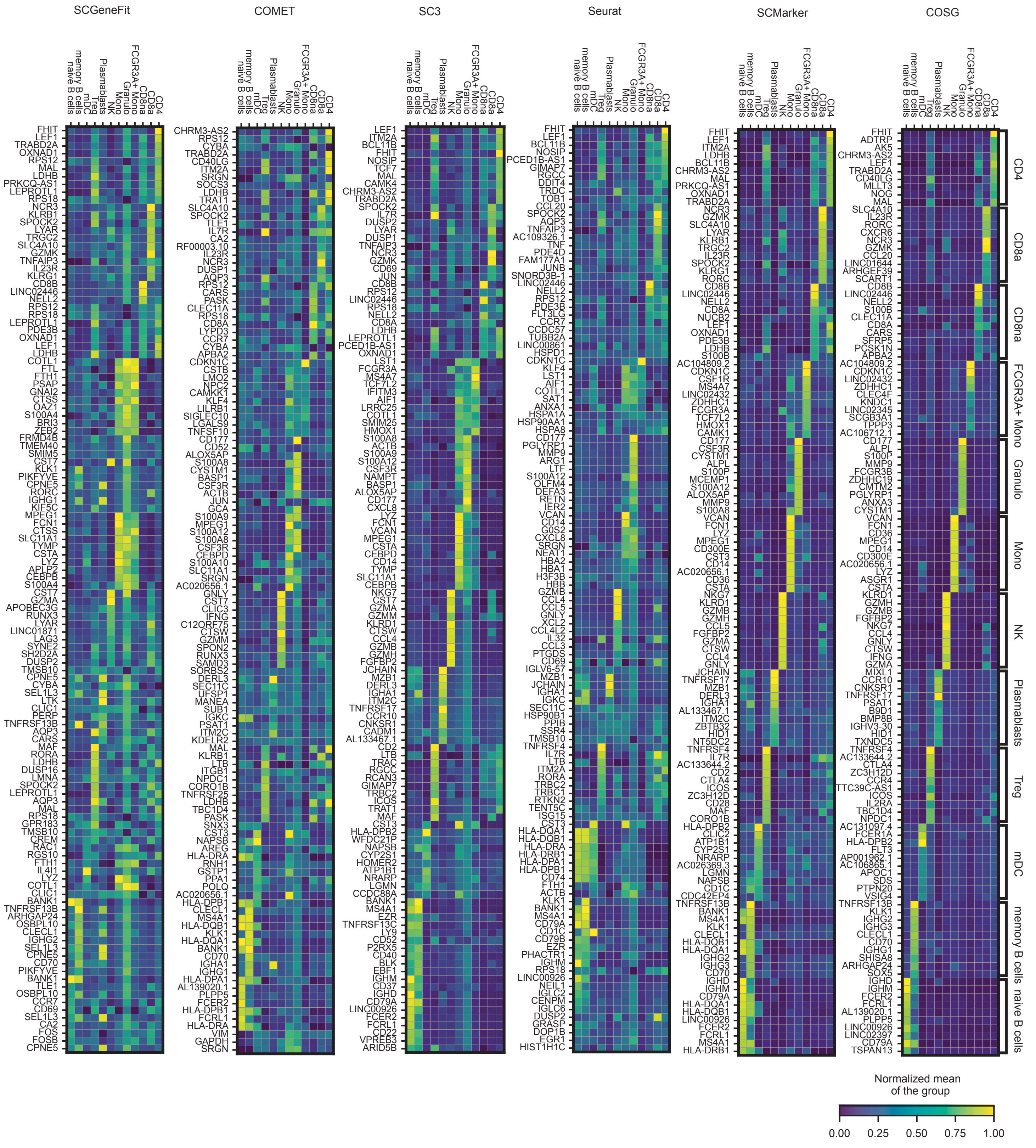

Supplementary Figure 11

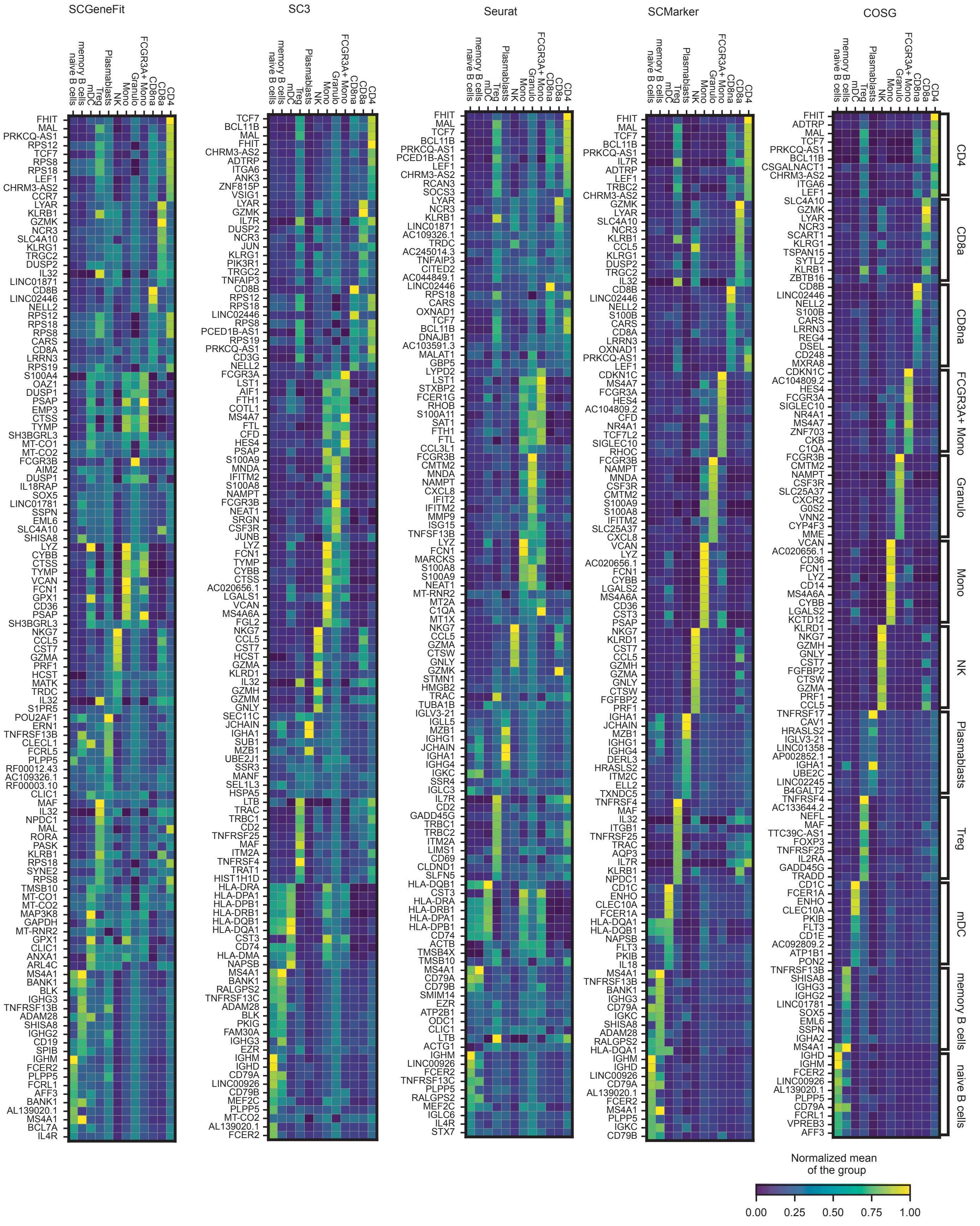

Supplementary Figure 11 (continued)

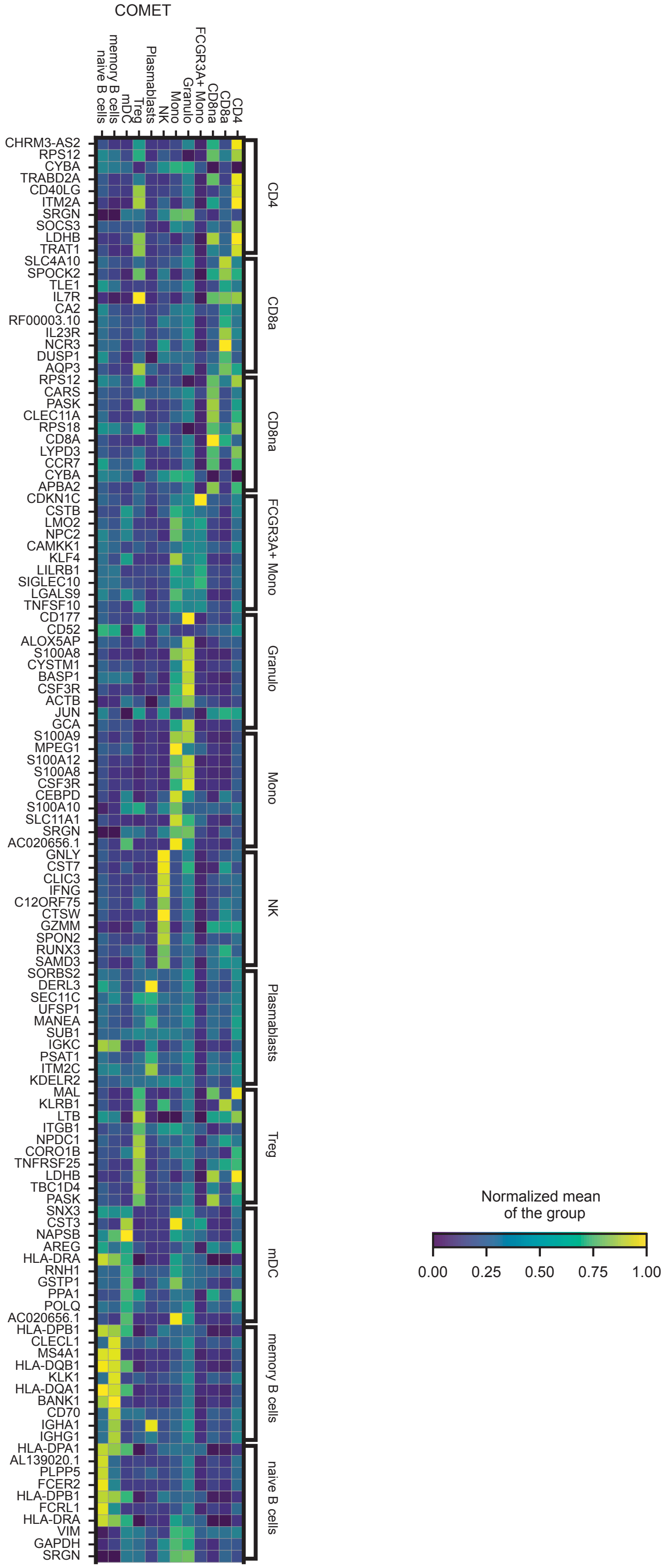

Supplement: Supplementary file 8 — Supplementary Figures. [file 41598_2024_63492_MOESM8_ESM.pdf]
